# Digitization of Follow-Up Care in Orthopedic and Trauma Surgery With Video Consultations: Health Economic Evaluation Study From a Health Provider’s Perspective

**DOI:** 10.2196/46714

**Published:** 2023-12-25

**Authors:** Jennifer Muschol, Martin Heinrich, Christian Heiss, Alher Mauricio Hernandez, Gero Knapp, Holger Repp, Henning Schneider, Ulrich Thormann, Johanna Uhlar, Kai Unzeitig, Christian Gissel

**Affiliations:** 1 Department of Health Economics Justus Liebig University Giessen Germany; 2 Department of Trauma, Hand and Reconstructive Surgery University Hospital Giessen Giessen Germany; 3 Bioinstrumentation and Clinical Engineering Research Group Bioengineering Department, Engineering Faculty Universidad de Antioquia Medellín Colombia; 4 Institute of Medical Informatics Justus Liebig University Giessen Germany

**Keywords:** digital health, economic evaluation, health economics, orthopedic, personnel costs, productivity gains, telemedicine, trauma surgery, utility, video consultations

## Abstract

**Background:**

Recommendations for health care digitization as issued with the Riyadh Declaration led to an uptake in telemedicine to cope with the COVID-19 pandemic. Evaluations based on clinical data are needed to support stakeholders’ decision-making on the long-term implementation of digital health.

**Objective:**

This health economic evaluation aims to provide the first German analysis of the suitability of video consultations in the follow-up care of patients in orthopedic and trauma surgery, investigate the financial impact on hospital operations and personnel costs, and provide a basis for decisions on digitizing outpatient care.

**Methods:**

We conducted a randomized controlled trial that evaluated video consultations versus face-to-face consultations in the follow-up care of patients in orthopedic and trauma surgery at a German university hospital. We recruited 60 patients who had previously been treated conservatively or surgically for various knee or shoulder injuries. A digital health app and a browser-based software were used to conduct video consultations. The suitability of telemedicine was assessed using the Telemedicine Satisfaction Questionnaire and the EQ-5D-5L questionnaire. Economic analyses included average time spent by physician per consultation, associated personnel costs and capacities for additional treatable patients, and the break-even point for video consultation software fees.

**Results:**

After 4 withdrawals in each arm, data from a total of 52 patients (telemedicine group: n=26; control group: n=26) were used for our analyses. In the telemedicine group, 77% (20/26) of all patients agreed that telemedicine provided for their health care needs, and 69% (18/26) found telemedicine an acceptable way to receive health care services. In addition, no significant difference was found in the change of patient utility between groups after 3 months (mean 0.02, SD 0.06 vs mean 0.07, SD 0.17; *P*=.35). Treatment duration was significantly shorter in the intervention group (mean 8.23, SD 4.45 minutes vs mean 10.92, SD 5.58 minutes; *P*=.02). The use of telemedicine saved 25% (€2.14 [US $2.35]/€8.67 [US $9.53]) in personnel costs and increased the number of treatable patients by 172 annually, assuming 2 hours of video consultations per week. Sensitivity analysis for scaling up video consultations to 10% of the hospital’s outpatient cases resulted in personnel cost savings of €73,056 (US $ 80,275.39) for a senior physician. A total of 23 video consultations per month were required to recoup the software fees of telemedicine through reduced personnel costs (break-even point ranging from 12-38 in the sensitivity analysis).

**Conclusions:**

Our study supports stakeholders’ decision-making on the long-term implementation of digital health by demonstrating that video consultations in the follow-up care of patients in orthopedic and trauma surgery result in cost savings and productivity gains for clinics with no negative impact on patient utility.

**Trial Registration:**

German Clinical Trials Register DRKS00023445; https://drks.de/search/en/trial/DRKS00023445

## Introduction

The adoption of digital technologies has progressed only gradually in health care systems, and uncertainty, especially with respect to the suitability and financial effects, has often acted as a drag on the broader use of digital health applications such as telemedicine [1-6]. The COVID-19 pandemic, however, has been transformative. Recommendations for health care digitization as issued with the Riyadh Declaration led to a strong increase in the use of telemedicine in medical specialties, including orthopedic and trauma surgery. The pandemic has boosted both demand for and supply of video consultations [1,3-5,7,8]. Under new pandemic rules, such as contact restrictions to contain infections, previous concerns about telemedicine have faded into the background, and the use of telemedicine appears likely to continue beyond the pandemic [1,7,9].

To support stakeholders’ decision-making on the long-term use of telemedicine in orthopedic and trauma surgery, analyses from a health provider’s perspective based on clinical data are required. Critical insights concerning the suitability of video consultations for patient care and the financial effects associated with telemedicine can be obtained by performing health economic evaluations.

This health economic evaluation aims to provide the first German analysis of the suitability of video consultations in the follow-up care of patients in orthopedic and trauma surgery, to investigate the associated financial and personnel impact, and to provide a basis for future decision-making on implementing telemedicine from a health provider’s perspective based on data from a randomized controlled trial (RCT). All economic analyses will be conducted from a health provider’s perspective, that is, from the perspective of the economic entity providing the health service. In this analysis, the economic entity providing follow-up care is a German university hospital. University hospitals provide the highest level of care in the German health care system and serve as important pioneers for establishing new standards of care.

Germany is the largest European health care market, with health expenditures of €457 billion (US $502.34 billion) in 2021 [10]. Despite its economic size, progress in health care digitization has been slow, with only 23% of adults having received a video consultation during the COVID-19 pandemic compared to a 45% average among Organization for Economic Cooperation and Development (OECD) countries [11]. Economic data from the health provider’s perspective showing the economic viability of telehealth remains a critical requirement for further diffusion of video consultations and other digital health care technologies.

Health economic evaluations of medical services and procedures, including telemedicine, are helpful at 2 distinct levels. First, on the macro level, health expenditures constitute a sizable part of spending for national economies. Data from the OECD show that OECD countries’ health care spending averaged about 8.8% of their gross domestic product before the COVID-19 pandemic in 2019. Individual countries, such as the United States at 16.8% and Germany at 11.7%, spent a significantly higher share on health [11]. It is estimated that during the COVID-19 pandemic, however, average health care spending as a share of the gross domestic product has already increased to 9.7% for 2020, and current forecasts indicate that the COVID-19 pandemic might further increase health expenditures in the long term [11,12]. Consequently, procedures and technologies that reduce costs and thus relieve the burden on health care systems are urgently needed. Only health economic evaluations can determine whether or not telemedicine holds this potential. Second, on the micro level, it is essential for various stakeholders, including hospitals and physicians, to know whether new procedures and technologies cause additional costs or promise to reduce costs while maintaining, or perhaps even increasing, patient utility. Only if new procedures are not inferior to conventional ones can their long-term implementation be recommended. Health economic evaluations thus serve to support stakeholders’ decision-making [13-15]. Apart from 2 Scandinavian studies, however, health economic evaluations that provide results from a health provider’s perspective in orthopedic and trauma surgery based on data from an RCT are limited [16,17].

In this analysis, we go beyond the health economic analysis of the RCT’s implemented scenario of 2 hours of video consultations per week. We extend our analysis with extensive calculations for scaling up video consultations in specific hospital departments as well as the entire hospital, analyzing the health economic effects of video consultations for 1%-10% of all patients who receive outpatient care at the hospital.

## Methods

### Study Design

We conducted an RCT to examine the use of telemedicine in the follow-up care of patients in orthopedic and trauma surgery at the University Hospital of Giessen, Germany, between September 2020 and April 2021. Our study design had 3 main goals: evaluation of patient and physician satisfaction, evaluation of economic and environmental impact from a societal perspective, and a health economic analysis of digitization from the hospital’s perspective. The first 2 evaluations have been previously published with a detailed description of our study design [18,19].

A total of 60 patients previously treated surgically or conservatively in the clinic for various shoulder and knee conditions were recruited for the RCT in the clinic or by telephone and were randomized in a 1:1 ratio. To participate in the study, patients had to be eligible to undergo a video consultation for their follow-up appointment. Patients in the intervention group (n=30) received a 1-time follow-up appointment through an online video consultation with their attending physician. The video consultation could be conducted by patients using a digital health app or a browser-based software. If a video consultation was not possible or if further diagnostics such as imaging were needed, patients could receive a face-to-face (F2F) appointment at any time. Patients in the control group (n=30) attended their follow-up appointment conventionally in the clinic.

In the Department of Trauma, Hand, and Reconstructive Surgery at the University Hospital of Giessen, a 1-hour time frame for video consultations was set up 2 days a week during regular clinic consultation hours as part of the RCT. Up to 8 telemedicine appointments were scheduled per week. Patients in both study arms were seen by the same senior physicians. These senior physicians used a laptop equipped with a camera and microphone to conduct the video consultations with a browser-based software. Although the technical equipment was already available in the clinic, additional costs in the form of monthly license fees for the use of the software occurred for the hospital during the study. Due to the simple design of the software, however, no training of the respective physicians and thus no training costs were required.

### Ethical Considerations

A detailed study protocol for the planned RCT was submitted and approved by the local ethics committee of the University of Giessen before the start of the study (AZ 73/20). Furthermore, the RCT was registered with the German Clinical Trials Register (DRKS00023445). Patients received comprehensive information about the study before participation and had to provide informed consent. No compensation was provided for participation in the study.

### Analysis of Telemedicine Suitability and its Economic Effects

The consideration of the health provider’s perspective comprised a bilateral analysis. In the first step, it was investigated whether telemedicine is suitable for hospitals in the follow-up care of patients in orthopedic and trauma surgery. As suggested by current literature, the investigation of video consultations’ suitability focused on the effectiveness of physician-patient communication and service provided in the form of a technology evaluation [4]. For this purpose, patients in the intervention group completed the Telemedicine Satisfaction Questionnaire (TSQ) by Yip et al [20], as this questionnaire evaluates the ability of telemedicine to meet the health care needs of patients [20]. Given that the TSQ was published in English, the questionnaire was translated into German with the help of the translation, review, adjunction, pretest, and documentation procedure, as recommended by the Leibniz Institute for the Social Sciences in Germany, during the preparation of the study [21]. The investigation of suitability furthermore included that patients in both groups completed the EQ-5D-5L questionnaire from the EuroQol Group, both at the time of recruitment and 3 months after recruitment, to assess differences in utility in terms of health-related quality of life between both groups [22]. The results of the first and second data collection of the EQ-5D-5L questionnaire were evaluated using the German EQ-5D-5L value set. The resulting utility values serve as a preference-based, health-related measure of quality of life and can range from –0.661 to 1. In this case, a utility value of 1 represents the best possible health status [23]. To avoid potential bias, utility was calculated only for patients who had completed both EQ-5D-5L questionnaires.

The descriptive analysis of the questionnaires included the presentation of the mean, SD, median, and relative frequencies. In addition, the Mann-Whitney *U* test was conducted to detect potential differences in the outcome of the EQ-5D-5L questionnaire between both groups.

In a second step, the economic effects of the use of video consultations were evaluated. These economic calculations comprised 4 different aspects with various sensitivity analyses and were guided by recommendations for health economic analyses in the context of eHealth interventions [24]. First, the time physicians spent on the respective consultations was compared between telemedicine and F2F consultations with the Mann-Whitney *U* test. The respective time difference was used to calculate personnel costs for both examination forms. As no additional support by nurses or other medical staff was required to perform the video consultations, the calculation of personnel costs focused exclusively on physicians’ salaries. More specifically, the hourly cost of a senior physician from the collective wage agreement for university hospitals was included in the calculation [25]. The use of publicly available data should ensure greater transparency and better transferability of the results. To increase this transparency and transferability, the personnel costs of deputy chief physicians, specialists, and assistant physicians were further considered in the cost calculation in the form of a sensitivity analysis. Second, model calculations were performed to consider the impact of expanding the number of video consultations on personnel cost savings. An expansion of video consultations was considered for different salaries and for both the respective department and the entire university hospital, with around 342,000 patients receiving outpatient care per year. Third, based on the time differences, the number of treatable patients was calculated and compared between telemedicine and F2F consultations. The number of additional treatable patients was further calculated by varying the weekly number of F2F consultations substituted by video consultations. Lastly, the break-even point of telemedicine was calculated by including personnel costs and software fees. For this purpose, the official monthly fee for unlimited use of the telemedicine software per physician was assessed [26]. Hospitals’ preexisting and readily available resources, including technical equipment (laptops with audio and video capabilities), an internet connection, and clinical premises, were not included in the cost calculation. Different assumptions were also made for the calculation of the break-even point in order to provide better transferability of the data. A sensitivity analysis included a lower software fee for a package that allows a maximum of 20 telemedical consultations per month and the salary of a deputy chief physician, a specialist, and an assistant physician rather than that of a senior physician [25,26].

## Results

### General Findings

The health economic evaluation was based on data from 26 patients in the intervention group and 26 patients in the control group after the withdrawal of 4 study participants in both treatment groups. In the telemedicine group, 42% (11/26) of participants were female, and 58% (15/26) were male. In addition, 27% (7/26) of participants in the telemedicine group were between 18 and 40 years of age, 65% (17/26) were between 41 and 60 years of age, and 8% (2/26) were aged 61 years or older. The reason for a follow-up appointment was a knee disorder in 38% (10/26) of cases and a shoulder disorder in 62% (16/26) of cases in the telemedicine group. In the control group, 38% (10/26) of patients were female, 62% (16/26) were male, 19% (5/26) were between 18 and 40 years of age, 58% (15/26) were between 41 and 60 years of age, and 23% (6/26) were aged 61 years or older. The medical indication of a knee disorder was given to 35% (9/26) of patients in the control group, and 65% (17/26) had a follow-up appointment due to a shoulder disorder. There were no significant differences between patient characteristics in both groups.

### Suitability of Telemedicine

The evaluation of the TSQ focused on the questions that evaluated physician-patient communication and the service provided and showed whether telemedicine is appropriate for use in clinical practice. These results are presented in Table 1.

**Figure 1:**
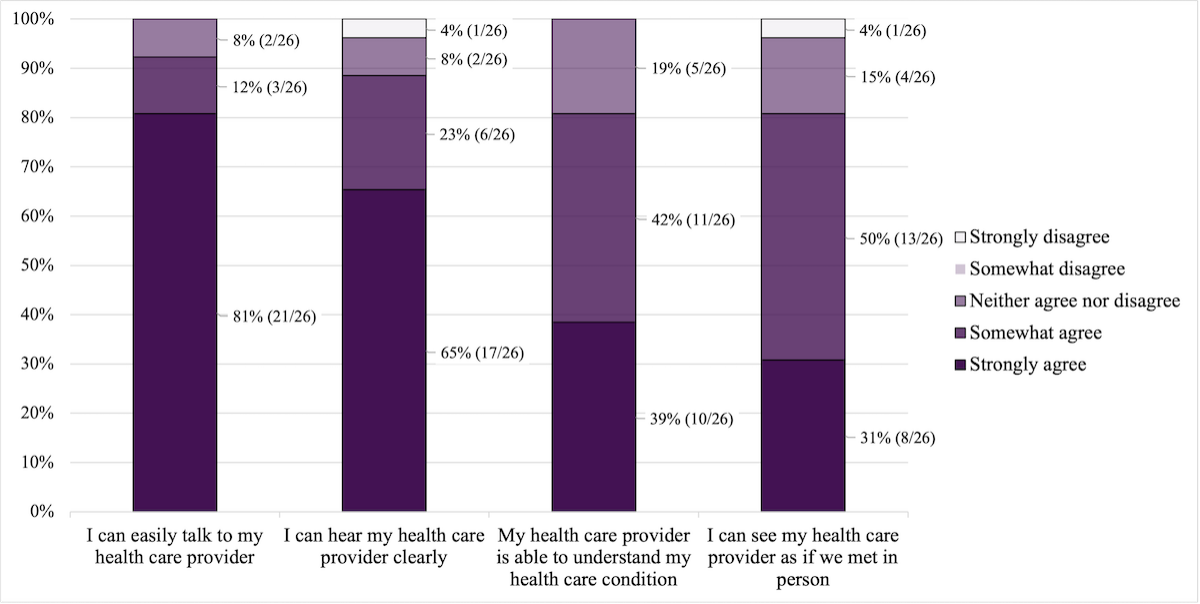
Distribution of the Telemedicine Satisfaction Questionnaire responses regarding physician-patient communication (n=26).

**Figure 2:**
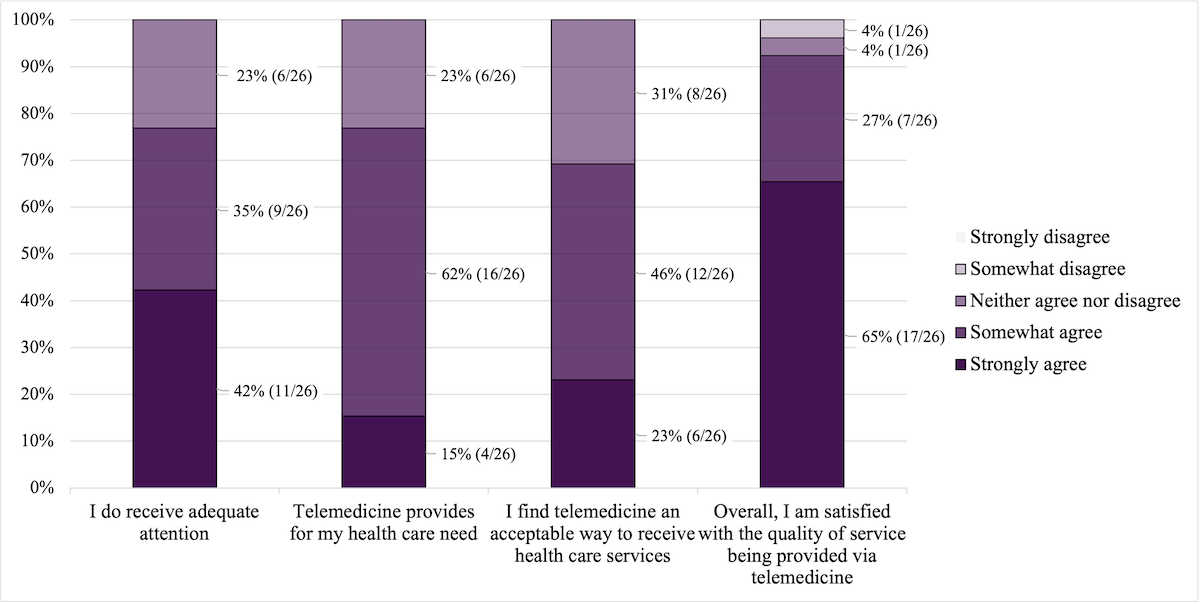
Distribution of the Telemedicine Satisfaction Questionnaire responses regarding service provided (n=26).

On a 5-point Likert scale ranging from 1 (strongly disagree) to 5 (strongly agree), the mean score for whether patients could easily talk to their health care provider was 4.73 (SD 0.60). The questions of whether patients could clearly hear their health care provider, whether the health care provider was able to understand the patients’ health care conditions, and whether patients could see their health care provider as if they were meeting in person were evaluated with mean scores of 4.46 (SD 0.95), 4.19 (SD 0.75), and 4.04 (SD 0.92), respectively. In addition, patients were asked to rate whether they received adequate attention through video consultations (mean 4.19, SD 0.80), whether telemedicine provided for their health care needs (mean 3.92, SD 0.63), whether they found telemedicine an acceptable way to receive health care services (mean 3.92, SD 0.74), and whether they were overall satisfied with the quality of service being provided through telemedicine (mean 4.54, SD 0.76). The distribution of the questions can be found in Figures 1 and 2.

The comparison of utility associated with health-related quality of life, as assessed by the German EQ-5D-5L value set, revealed no significant differences between both groups, either at baseline or after 3 months, as shown in Table 2.

**Table 1:** Suitability of telemedicine.

Telemedicine group (n=26)	Mean^a^ (SD)	Median^a^ (IQR)
I can easily talk to my health care provider	4.73 (0.60)	5.00 (5-5)
I can hear my health care provider clearly	4.46 (0.95)	5.00 (4-5)
My health care provider is able to understand my health care condition	4.19 (0.75)	4.00 (4-5)
I can see my health care provider as if we met in person	4.04 (0.92)	4.00 (4-5)
I do receive adequate attention	4.19 (0.80)	4.00 (3.75-5)
Telemedicine provides for my health care need	3.92 (0.63)	4.00 (3.75-4)
I find telemedicine an acceptable way to receive health care services	3.92 (0.74)	4.00 (3-4.25)
Overall, I am satisfied with the quality of service being provided through telemedicine	4.54 (0.76)	5.00 (4-5)

^a^5-point Likert scale; from 1=strongly disagree to 5=strongly agree.

**Table 2:** Comparison of utility values between groups.

Variables	Telemedicine group (n=26)					Control group (n=26)				*P* value^a^
	Participants, n (%)	Mean (SD)		Median (IQR)	Participants, n (%)		Mean (SD)	Median (IQR)		
Utility value at baseline	16 (62)	0.80 (0.19)		0.88 (0.77-0.91)	14 (54)		0.74 (0.27)	0.85 (0.70-0.89)	.36	
Utility value after 3 months	16 (62)	0.82 (0.19)		0.89 (0.81-0.91)	14 (54)		0.81 (0.23)	0.87 (0.79-0.92)	.94	
∆ utility value	16 (62)	0.02 (0.06)		0.00 (0.00-0.04)	14 (54)		0.07 (0.17)	0.03 (0.01-0.08)	.35	

^a^*P* values were based on the Mann-Whitney *U* test.

The mean utility values at baseline were 0.80 in the telemedicine group and 0.74 in the control group (*P*=.36). After 3 months, the utility values increased to 0.81 for the telemedicine group and 0.82 for the control group (*P*=.94). Although this utility increase in the control group, at 0.07, was stronger than that in the telemedicine group, at 0.02, the difference in change between the groups was not statistically significant (*P*=.35).

### Economic Effects

The economic effects of video consultations for the follow-up care of patients in orthopedic and trauma surgery from the health provider’s perspective comprised several calculations. First, the treatment duration that was used for the health economic calculations showed a significant difference between both groups. In the intervention group, the treatment duration, at an average of 8.23 (SD 4.45; median 6.00, IQR 5-10) minutes, was significantly shorter than that in the control group (average 10.92, SD 5.58 minutes; median 10.00, IQR 8.0-14.5 minutes; *P*=.02). Based on the salary of a senior physician, a video consultation resulted in average personnel costs of €6.54 (US $7.19) and an F2F consultation in personnel costs of €8.67 (US $9.53). The time saving of 2.69 minutes between both groups corresponded to a saving of €2.14 (US $2.35) in personnel costs for each telemedicine appointment, compared with an in-clinic one; that is, with the help of telemedicine, 25% (€2.14 [US $2.35]/€8.67 [US $9.53]) of personnel costs could be saved. Table 3 shows the personnel costs for different physician salaries in the context of the sensitivity analysis. Savings in personnel costs ranged from €1.29 (US $1.42) to €2.51 (US $2.76) per video consultation.

Second, when treating 8 patients through telemedicine in 2 consultation hours per week, as was the case in this study, this would result in savings of €820.28 (US $901.67) per year in personnel costs for senior physicians. The sensitivity analysis showed that the savings ranged from €496.18 (US $545.41) to €964.92 (US $1060.66) per year for the different salaries, as can be seen in Table 4.

If video consultations were expanded to all 6 specialty consultation hours of the department with 24 patients per week, the annual savings in personnel costs would be €2460.84 (US $2705.00) for a senior physician, ranging from €1488.55 (US $1636.24) to €2894.76 (US $3181.98).

In addition, if telemedicine were expanded to more departments and about 1% (n=3420) of the 342,000 patients who receive outpatient care at the university hospital were treated by video consultations per year, €7305.62 (US $8030.48) of personnel costs could be saved for senior physicians (ranging from €4419.14 [US $4857.61] to €8593.81 [US $ 9446.49] in the sensitivity analysis). At 5% (17,100/342,000) and 10% (34,200/342,000) of all outpatient cases, respectively, the personnel costs saved would increase to €36,528.09 [US $40,152.41] and €73,056.18 [US $80,304.81] for senior physicians, ranging from €22,095.72 [US $24,288.06] to €85,938.09 [US $94,464.87].

Third, with an average treatment duration of 8.23 minutes per patient in the intervention group, around 7.29 patients per hour could be treated on average through a video consultation. The average treatment duration of 10.92 minutes for patients in the control group leads to an average of 5.49 treatable patients based on F2F consultations. If 2 hours of F2F consultations per week were substituted by video consultations, 172.41 additional patients could be treated annually, as shown in Table 5.

If 5 hours were substituted, the number of additionally treatable patients could increase to 431.01, and if 10 hours were substituted, the number could increase to 862.03 additional patients per year.

Lastly, the monthly fee for unlimited use of the telemedicine software was €49.00 (US $53.86) per physician. This resulted in a break-even point of 22.94, meaning that the costs of telemedicine would be recouped through savings in personnel costs after 23 telemedicine consultations per month and physician. A lower software fee of €29 (US $31.88) would lower the break-even point to 13.58 telemedical consultations per month and physician. In this case, however, the health provider’s profit margin would be capped because the lower software fee entails an upper limit of 20 telemedical consultations per month. Table 6 shows that the break-even point ranged from 11.54 to 37.92 for the different software fees and salaries. Multimedia Appendix 1 presents a detailed presentation of model calculations.

**Table 3:** Analysis of personnel costs. A currency exchange rate of €1=US $1.10 is applicable.

Personnel costs	Video consultation (€)	F2F^a^ consultation (€)	Difference (€)
Senior physician	6.54	8.67	2.14
Deputy chief physician	7.69	10.20	2.51
Specialist	5.22	6.92	1.71
Assistant physician	3.95	5.25	1.29

^a^F2F: face-to-face.

**Table 4:** Analysis of the substitution of face-to-face (F2F) consultations with video consultations. A currency exchange rate of €1=US $1.10 is applicable.

Substituted F2F consultations			Saved personnel costs (€)
**2 consultation hours per week**			
	Senior physician	820.28	
	Deputy chief physician	964.92	
	Specialist	654.88	
	Assistant physician	496.18	
**6 consultation hours per week**			
	Senior physician	2460.84	
	Deputy chief physician	2894.76	
	Specialist	1964.65	
	Assistant physician	1488.55	
**1% (3420/342,000) of patients who receive outpatient care at the clinic**			
	Senior physician	7305.62	
	Deputy chief physician	8593.81	
	Specialist	5832.56	
	Assistant physician	4419.14	
**5% (17,100/342,000) of patients who receive outpatient care at the clinic**			
	Senior physician	36,528.09	
	Deputy chief physician	42,969.04	
	Specialist	29,162.82	
	Assistant physician	22,095.72	
**10% (34,200/342,000) of patients who receive outpatient care at the clinic**			
	Senior physician	73,056.18	
	Deputy chief physician	85,938.09	
	Specialist	58,325.64	
	Assistant physician	44,191.44	

**Table 5:** Analysis of additional patients treatable when substituting face-to-face (F2F) consultations with video consultations.

Substituted F2F consultations	Additional patients treatable, n
2 hours of video consultations per week	172.41
5 hours of video consultations per week	431.01
10 hours of video consultations per week	862.03

**Table 6:** Analysis of the break-even point (number of telemedicine consultations per month and physician). A currency exchange rate of €1=US $1.10 is applicable.

Type of physician and amount of software fee	Break-even point
Senior physician (€49)	22.94
Senior physician (€29)	13.58
Deputy chief physician (€49)	19.50
Deputy chief physician (€29)	11.54
Specialist (€49)	28.73
Specialist (€29)	17.00
Assistant physician (€49)	37.92
Assistant physician (€29)	22.44

## Discussion

### Principal Results

This health economic analysis from a health provider’s perspective showed important insights for stakeholder decision-making on the long-term use of telemedicine in the follow-up care of patients in orthopedic and trauma surgery by examining both the suitability of video consultations and the associated financial and personnel effects.

The results of the TSQ indicated that the majority of patients positively evaluated the physician-patient communication and service provided through video consultations. These results are similar to findings in other surgical specialties [27,28].

Although video consultations were 25% (2.69/10.92 minutes) shorter than F2F consultations, there was no significant difference in patient utility regarding health-related quality of life between the telemedicine group and the control group. In a former study, we already compared the EQ-visual analog scale between the intervention and the control group [18]. In this study, we furthermore considered the responses of the EQ-5D descriptive system and evaluated them using the German value set in order to analyze whether significant differences between the groups occurred. The comparison shows that no significant differences were found between video consultations and F2F consultations, neither for the visual analog scale nor for the descriptive system based on the German value set. Thus, it could be argued that telemedicine can save costs while maintaining patient utility—a finding supported by Buvik et al [29], who were also unable to show relevant differences in EQ-5D-assessed patient utility between telemedical and F2F follow-ups [29]. At the same time, it is important to monitor the use and implementation of telemedicine so as to ensure that patient utility is not negatively affected by shortening treatment duration in the long run. Nonetheless, the fact that a video consultation is less time-consuming for physicians than a clinical consultation is also confirmed in an RCT of telemedicine in the follow-up of arthroscopic rotator cuff surgery conducted by Kane et al [30], who also did not find any negative patient outcomes associated with the performance of video consultations [30].

From the health provider’s perspective, these results suggest that video consultations might be suitable for use in orthopedic and trauma surgery. The potential of video consultations is further underlined by previous studies that found comparable results to F2F consultations in terms of physician and patient satisfaction, efficiency, quality of care, and benefits from a societal perspective [16,18,19,29-32].

The economic impact of using telemedicine can be differentiated for clinics both as providers of medical services and as employers. As providers of medical services, clinics benefit from productivity gains due to a reduced consultation time, which on the one hand could lead to lower personnel costs and thus relieve the burden on the health care system, and on the other hand could result in an increased capacity of a clinic and thus improve patient care through shorter waiting times and mitigate the shortage of physicians in the health care system [33,34]. In addition, the implementation of telemedicine could result in a competitive advantage for clinics, as communication with patients is simplified and, as a result, a service beyond the local environment could be offered [35]. As telemedicine is associated with cost and time savings for patients as well, the offering of video consultations could furthermore help to recruit new patients [16,17,19,30]. The implementation of video consultations could also create the possibility of a home office for physicians. The resulting benefit of the clinic as an employer could be a competitive advantage in personnel recruitment as well as an increase in the satisfaction of the permanent personnel, as studies indicate an improved work-life balance associated with working from home [36,37]. Alternative working arrangements are especially attractive for all physicians taking care of a family. In its 2018 policy tag on work-life balance, the World Medical Association argued for the promotion of inclusiveness through gender equality. In particular, the World Medical Association encouraged more efforts to explore telecommunication opportunities to allow for more flexibility in balancing the work-life demands of physicians [38].

A holistic view of the economic effects of telemedicine, however, must consider not only the cost savings but also the additional costs incurred by the clinic as a result of the technology.

A minimum of 23 video consultations per physician per month was required to recoup the costs of investing in telemedicine software through a reduction in personnel costs resulting from time savings. In the sensitivity analysis, the break-even point ranged from 11.54 to 37.92 video consultations. A lower software fee, however, effectively capped the number of video consultations at 20 per month. Whether this is a viable option for decision makers in practice depends on their individual objectives. Competing providers of telemedicine software in Germany may well offer lower fees that would help lower the break-even point. A given hospital’s possibility of negotiating individual terms of use and fee structures with telemedicine providers might be another important aspect to take into account when implementing telemedicine. Finally, physicians’ incomes are rising continuously. The calculations of personnel costs were based on the cost rates in effect at the time the study was conducted. In 2023, salaries will increase by up to 5.13%. The savings of higher personnel costs through telemedicine will then be accompanied by a lower break-even point. The break-even points calculated in earlier contributions by Buvik et al [16] (183 telemedicine consultations per year from the health provider perspective and 151 from the societal perspective) and Ohinmaa et al [17] (80 consultations from the societal perspective) cannot be directly compared with the break-even point arrived at in our analysis. Their studies (1) focused on telemedicine provided with the help of a local caregiver rather than independently of location, and (2) featured other aspects in their cost calculations [16,17].

This health economic evaluation provides clinical evidence on the apparent ability of telemedicine to provide similar patient utility at lower cost and can therefore improve stakeholders’ decisions on implementing telemedicine in the follow-up care of patients in orthopedic and trauma surgery both in and beyond the current COVID-19 pandemic [39]. The potential transferability of these findings to other medical specialties due to the practical study design has high practical relevance, particularly in light of rising health care expenditures and ongoing shortages of physicians [12,33].

### Limitations

There are 3 main limitations. On the one hand, the treatment duration was not measured precisely but was collected from the physicians by means of a questionnaire. However, individual deviations should compensate for each other in total. Furthermore, the technical equipment, the internet connection, and the clinical premises were not considered in the overall cost analysis, as these were already available and did not cause any additional costs for the clinic. In the case of a transfer to other clinics, these costs should be examined in advance. Another limitation arises from the response rates to the 2 data collections of the EQ-5D-5L questionnaire: only patients who had completed both questionnaires were included in the calculation to avoid potential bias. As a result, the group sizes considered were limited.

### Conclusions

The first health economic evaluation based on data from a German RCT demonstrated that the use of telemedicine might be suitable for the follow-up care of patients in orthopedic and trauma surgery regarding the physician-patient communication and service provided and that video consultations are less time-consuming for physicians compared with conventional F2F consultations, resulting in personnel cost savings and productivity gains for clinics without any negative impact on patient utility. These findings were collected from the health provider’s perspective and can thus provide targeted support to stakeholders in decision-making on the long-term use of telemedicine. Results might differ for other specialties of surgery and for different implementations of digital health, such as remote monitoring. However, our results could be helpful in estimating the effects before the implementation of digital processes in other specialties.

## Appendix

Multimedia Appendix 1 Detailed presentation of cost calculations.

## Abbreviations

F2F: face-to-face
OECD: Organization for Economic Cooperation and Development
RCT: randomized controlled trial
TSQ: Telemedicine Satisfaction Questionnaire

## Data Availability

The data sets generated and analyzed during this study are available from the corresponding author on reasonable request.
