# Supplementary material for: Digitization of Follow-Up Care in Orthopedic and Trauma Surgery With Video Consultations: Health Economic Evaluation Study From a Health Provider’s Perspective
Source: J Med Internet Res. 2023 Dec 25;25:e46714. doi: 10.2196/46714 (PMC10775022; doi:10.2196/46714)
Supplement: Multimedia Appendix 1 [file jmir_v25i1e46714_app1.docx]

| Multimedia Appendix 1: Detailed presentation of cost calculations | | | | | |
| --- | --- | --- | --- | --- | --- |
|  | Senior physician | Deputy chief physician | Specialist | Assistant physician |  |
| Personnel costs per video consultation | €8,004.59 / 168 hours / 60 minutes x 8.23 minutes = €6.54 | €9,416.03 / 168 hours / 60 minutes x 8.23 minutes = €7.69 | €6,390.60 / 168 hours / 60 minutes x 8.23 minutes = €5.22 | €4,841.95/ 168 hours / 60 minutes x 8.23 minutes = €3.95 |  |
| Personnel costs per F2F consultation | €8,004.59 / 168 hours / 60 minutes x 10.92 minutes = €8.67 | €9,416.03 / 168 hours / 60 minutes x 10.92 minutes = €10.20 | €6,390.60 / 168 hours / 60 minutes x 10.92 minutes = €6.92 | €4,841.95/ 168 hours / 60 minutes x 10.92 minutes = €5.25 |  |
| Substitution of two consultation-hours (8 patients) per week with video consultations | 384 patients x (€8,004.59 / 168 hours / 60 minutes x 10.92 minutes) - 384 patients x (€8,004.59 / 168 hours / 60 minutes x 8.23 minutes) = €820.28 | 384 patients x (€9,416.03 / 168 hours / 60 minutes x 10.92 minutes) - 384 patients x (€9,416.03 / 168 hours / 60 minutes x 8.23 minutes) = €964.92 | 384 patients x (€6,390.60 / 168 hours / 60 minutes x 10.92 minutes) - 384 patients x (€6,390.60 / 168 hours / 60 minutes x 8.23 minutes) = €654.88 | 384 patients x (€4,841.95 / 168 hours / 60 minutes x 10.92 minutes) - 384 patients x (€4,841.95/ 168 hours / 60 minutes x 8.23 minutes) = €496.18 |  |
| Substitution of six consultation-hours (24 patients) per week with video consultations | 1,152 patients x (€8,004.59 / 168 hours / 60 minutes x 10.92 minutes) - 1,152 patients x (€8,004.59 / 168 hours / 60 minutes x 8.23 minutes) = €2,460.84 | 1,152 patients x (€9,416.03 / 168 hours / 60 minutes x 10.92 minutes) - 1,152 patients x (€9,416.03 / 168 hours / 60 minutes x 8.23 minutes) = €2,894.76 | 1,152 patients x (€6,390.60 / 168 hours / 60 minutes x 10.92 minutes) - 1,152 patients x (€6,390.60 / 168 hours / 60 minutes x 8.23 minutes) = €1,964.65 | 1,152 patients x (€4,841.95 / 168 hours / 60 minutes x 10.92 minutes) - 1,152 patients x (€4,841.95/ 168 hours / 60 minutes x 8.23 minutes) = €1,488.55 |  |
| 1% of ambulatory patients in the clinic treated via video consultations | 342,000 patients x 0.01 x (€8,004.59 / 168 hours / 60 minutes x 10.92 minutes) - 342,000 patients x 0.01 x (€8,004.59 / 168 hours / 60 minutes x 8.23 minutes) = €7,305.62 | 342,000 patients x 0.01 x (€9,416.03 / 168 hours / 60 minutes x 10.92 minutes) - 342,000 patients x 0.01 x (€9,416.03 / 168 hours / 60 minutes x 8.23 minutes) = €8,593.81 | 342,000 patients x 0.01 x (€6,390.60 / 168 hours / 60 minutes x 10.92 minutes) - 342,000 patients x 0.01 x (€6,390.60 / 168 hours / 60 minutes x 8.23 minutes) = €5,832.56 | 342,000 patients x 0.01 x (€4,841.95 / 168 hours / 60 minutes x 10.92 minutes) - 342,000 patients x 0.01 x (€4,841.95/ 168 hours / 60 minutes x 8.23 minutes) = €4,419.14 |  |
| 5% of ambulatory patients in the clinic treated via video consultations | 342,000 patients x 0.05 x (€8,004.59 / 168 hours / 60 minutes x 10.92 minutes) - 342,000 patients x 0.05 x (€8,004.59 / 168 hours / 60 minutes x 8.23 minutes) = €36,528.09 | 342,000 patients x 0.05 x (€9,416.03 / 168 hours / 60 minutes x 10.92 minutes) - 342,000 patients x 0.05 x (€9,416.03 / 168 hours / 60 minutes x 8.23 minutes) = €42,969.04 | 342,000 patients x 0.05 x (€6,390.60 / 168 hours / 60 minutes x 10.92 minutes) - 342,000 patients x 0.05 x (€6,390.60 / 168 hours / 60 minutes x 8.23 minutes) = €29,162.82 | 342,000 patients x 0.05 x (€4,841.95 / 168 hours / 60 minutes x 10.92 minutes) - 342,000 patients x 0.05 x (€4,841.95/ 168 hours / 60 minutes x 8.23 minutes) = €22,095.72 |  |
| 10% of ambulatory patients in the clinic treated via video consultations | 342,000 patients x 0.1 x (€8,004.59 / 168 hours / 60 minutes x 10.92 minutes) - 342,000 patients x 0.1 x (€8,004.59 / 168 hours / 60 minutes x 8.23 minutes) = €73,056.18 | 342,000 patients x 0.1 x (€9,416.03 / 168 hours / 60 minutes x 10.92 minutes) - 342,000 patients x 0.1 x (€9,416.03 / 168 hours / 60 minutes x 8.23 minutes) = €85,938.09 | 342,000 patients x 0.1 x (€6,390.60 / 168 hours / 60 minutes x 10.92 minutes) - 342,000 patients x 0.1 x (€6,390.60 / 168 hours / 60 minutes x 8.23 minutes) = €58,325.64 | 342,000 patients x 0.1 x (€4,841.95 / 168 hours / 60 minutes x 10.92 minutes) - 342,000 patients x 0.1 x (€4,841.95/ 168 hours / 60 minutes x 8.23 minutes) = €44,191.44 |  |
| Break-even point €49.00 software fee | €49 / (10.92 minutes x (€8,004.59 / 168 hours / 60 minutes) - (8.23 minutes x (€8,004.59 / 168 hours / 60 minutes))) = 22.94 | €49 / (10.92 minutes x (€9,416.02 / 168 hours / 60 minutes) - (8.23 minutes x (€9,416.03 / 168 hours / 60 minutes))) = 19.50 | €49 / (10.92 minutes x (€6,390.60 / 168 hours / 60 minutes) - (8.23 minutes x (€6,390.60 / 168 hours / 60 minutes))) = 28.73 | €49 / (10.92 minutes x (€4,841.95 / 168 hours / 60 minutes) - (8.23 minutes x (€4,841.95 / 168 hours / 60 minutes))) = 37.92 |  |
| Break-even point €29.00 software fee | €29 / (10.92 minutes x (€8,004.59 / 168 hours / 60 minutes) - (8.23 minutes x (€8,004.59 / 168 hours / 60 minutes))) = 13.58 | €29 / (10.92 minutes x (€9,416.02 / 168 hours / 60 minutes) - (8.23 minutes x (€9,416.03 / 168 hours / 60 minutes))) = 11.54 | €29 / (10.92 minutes x (€6,390.60 / 168 hours / 60 minutes) - (8.23 minutes x (€6,390.60 / 168 hours / 60 minutes))) = 17.00 | €29 / (10.92 minutes x (€4,841.95 / 168 hours / 60 minutes) - (8.23 minutes x (€4,841.95 / 168 hours / 60 minutes))) = 22.44 |  |
|  | | | | | |
| Treatable patients | Video consultation: 60 minutes / 8.23 minutes = 7.29 patients per hour F2F consultation: 60 minutes / 10.92 minutes = 5.49 patients per hour  2 hours of video consultations per week: 96 hours x (60 minutes / 8.23 minutes) - 96 hours x (60 minutes / 10.92 minutes) = 172.41 patients  5 hours of video consultations per week: 240 hours x (60 minutes / 8.23 minutes) - 240 hours x (60 minutes / 10.92 minutes) = 431.01 patients  10 hours of video consultations per week: 480 hours x (60 minutes / 8.23 minutes) - 480 hours x (60 minutes / 10.92 minutes) = 862.03 patients | | | | |
